# Supplementary material for: Machine learning assisted DSC-MRI radiomics as a tool for glioma classification by grade and mutation status
Source: BMC Med Inform Decis Mak. 2020 Jul 6;20:149. doi: 10.1186/s12911-020-01163-5 (PMC7336404; doi:10.1186/s12911-020-01163-5)
Supplement: Supplementary file 1 — Additional file 1: Table S1. Demographic details on the population. Table S2. Cliff’s Delta obtained for the comparison of each feature (intensity and shape with respect to the different acquisition parameters. Table S3. Analysis of error and absolute errror in the prediction of IDH status and Grade according to acquisition parameters.Table S4. Confusion matrix over different feld strengths for the mutation status prediction(top row) and the grade prediction(bottom row). Table S5. For all error types, mean z-score difference between the erroneously classified elements and the rightly classifed elements for each feature corrected for acquisition parameters. Figure S1. Representation of the acquisition parameters repartition across the 333 used cases. [file 12911_2020_1163_MOESM1_ESM.pdf]

Supplementary Material

Expression of Haralick features

In order to create the texture features the normalised intensities are quantised in .. bins and the rotational invariant matrix P of intensity cooccurrence over tumor mask M of size NxN is computed as follows:

$$P(i,j) = 16 * N^2 \sharp(r_1 = (x_1, y_1, z_1), r_2 = (x_2, y_2, z_2)) || r_1 - r_2|_{L1} == 1 and I(r_1) = i and I(r_2) = j$$

From the co-occurence matrix, the following notations are used:
The 14 Haralick features are then expressed as:

$p_x(i) = \sum_{j=1}^{N} p(i,j)$	$p_y(j) = \sum_{i=1}^{N} p(i,j)$	$\mu_x = 1N \sum_{i=1}^{N} p_x(i)$	$\mu_y = 1N \sum_{j=1}^{N} p_y(j)$
$p_{x-y}(k) = \|i-j\| = k \sum_{i=1}^{N} \sum_{j=1}^{N} p(i,j)$	$p_{x+y}(k) = \|i+j\| = k \sum_{i=1}^{N} \sum_{j=1}^{N} p(i,j)$	$\sigma_x = \sqrt{\sum_i (p_x(i) - \mu_x)^2 N - 1}$	$\sigma_y = \sqrt{\sum_j (p_y(j) - \mu_y)^2 N - 1}$
$HXY = -\sum_i \sum_j p(i,j) \log(p(i,j)$	$HX = -\sum_i p_x(i) \log(p_x(i))$	$HY = -\sum_j p_y(j) \log(p_y(j))$	
$HXY1 = -\sum_i \sum_j p(i,j) \log(p_x(i) p_y(j))$		$HXY2 = -\sum_i \sum_j p_x(i) p_y(j) \log(p_x(i) p_y(j))$	

Angular Second Moment $\sum_i \sum_j p(i,j)^2$

Contrast $\sum_n n^2 (p_{x-y}(n))$

Correlation $\sum_i \sum_j ijp(i,j) - \mu_x \mu_y \sigma_x \sigma_y$

Sum of squares $\sum_i \sum_j (i - \mu)^2 p(i,j)$

Inverse Difference Moment $\sum_i \sum_j 11 - (i-j)^2 p(i,j)$

Sum Average $\sum_n^{2N} n (p_{x+y}(n))$

Entropy $-\sum_i \sum_j p(i,j) \log p(i,j)$

Sum entropy $-\sum_i p_{x+y}(i) \log p_{x+y}(i)$

Difference variance $1N - 1 \sum_n (p_{x-y}(i) - \mu_{x-y})^2$

Difference entropy $\sum_i p_{x-y}(i) \log(p_{x-y}(i)$

Sum Variance $\sum_i (i - SE)^2 p_{x+y}(i)$

Information measures of correlation1 $HXY - HXY1max(HX, HY)$

Information measures of correlation 2 $\sqrt{1 - \exp(-2 * HXY2 - HXY)}$

Supplementary Tables

Table S 1: Demographic details on the population

		Grade II		Grade III		Grade IV		
		WT	IDH	WT	IDH	WT	IDH	Total
Number		14	87	27	47	141	17	333
Age - Mean (SD)		39.2 (14.4)	40.1 (12)	51.6 (13.9)	43.7 (12.6)	56.4 (12.8)	48.9 (14.9)	48.9 (14.6)
Gender	Female / Male	3 / 11	33 / 54	19 / 8	17 / 30	56 / 85	7 / 10	135 / 198
Center	1	5	42	10	15	48	7	127
	2	0	14	0	14	32	3	63
	3	4	13	4	4	10	1	36
	4	2	6	8	3	23	0	42
	5	3	11	3	9	23	1	50
	6	0	1	2	2	5	5	15
Pathology	Glioblastoma/ Oligoden-droglioma	11 / 3	54 / 33	22 / 5	25 / 22	141 / 0	17 / 0	270 / 63
Tumour side	Left / Right /Both	1/13/0	36/49/2	7/17/3	20/25/2	66/60/15	5/10/2	135/174/24
Tumour lobe	Frontal	4	41	12	28	57	6	148
	Temporal	3	19	6	6	28	3	65
	Insular	0	13	1	2	4	1	21
	Frontotemporal	1	6	3	4	7	0	21
	Parietal	0	4	0	2	8	2	16
	Corpus callosum	0	0	0	1	5	1	7
	MultiLobe	4	4	3	4	24	4	43
	Occipital	0	0	0	0	4	0	4
	Brainstem / Cerebellum	2	0	2	0	4	0	8

Table S 2: Cliff's Delta obtained for the comparison of each feature (intensity and shape with respect to the different acquisition parameters. The groups for each acquisition parameters are as follows: FA: Flip angle 0 - 90; 1 - <90; FS: Field Strength 0 - 1.5T / 1 - 3T; MAT: Matrix Acquisition 0 - $<$ 128 X 128 / 1 - $>$ 128X128; RES: Axial resolution 0 - ¡= 1mm X 1mm / 1 [1mm - 2mm] / 2 - $>$ 2mm; SCAN: Scanner manufacturer 0 SIEMENS / 1 Philips / 2 GE; SEQ: Sequence acquisition 0 Spin echo / 1 Gradient Echo; SLICE: Slice thickness 0 $<$ 5mm / 1 $\geq$ 5mm ; TE : Echo time 0 - [25 - 44] ms / 1 - [45 - 55 ms] ; TR Repetition time 0 - <1499ms / 1 - $>$ 1500ms

	**FA_01**	**FS_01**	**MAT_01**	**RES_01**	**RES_02**	**RES_12**	**SCAN_01**	**SCAN_02**	**SCAN_12**	**SEQ_01**	**SLICE_01**	**TE_01**	**TR_01**
**Mean**	0.073	0	0.173	0.041	-0.129	-0.167	-0.239	0.049	0.301	-0.123	-0.032	-0.129	-0.096
**Skewness**	-0.206	0.099	-0.292	-0.22	0.036	0.257	0.269	-0.347	-0.576	0.077	-0.195	0.134	0.176
**Kurtosis**	-0.21	0.117	-0.288	-0.252	0.009	0.279	0.257	-0.35	-0.587	0.077	-0.196	0.147	0.199
**Min**	0.037	0.145	0.164	0.151	-0.005	-0.177	-0.159	-0.282	-0.203	-0.157	-0.202	-0.221	-0.052
**Max**	-0.224	-0.116	-0.398	-0.467	-0.254	0.275	0.165	-0.17	-0.368	0.03	-0.113	0.177	0.273
**STD**	0.071	-0.09	0.045	0.027	-0.014	-0.058	-0.1	0.12	0.222	-0.052	0.036	-0.104	-0.104
**P1**	-0.068	0.123	0.018	-0.02	-0.102	-0.072	-0.089	-0.291	-0.271	-0.118	-0.255	-0.05	0.048
**P5**	-0.089	0.093	0	-0.156	-0.204	-0.031	-0.115	-0.235	-0.136	-0.107	-0.202	0.028	0.045
**P25**	-0.005	0.077	0.126	-0.087	-0.161	-0.089	-0.2	-0.053	0.153	-0.101	-0.101	-0.124	-0.05
**P50**	0.068	0.044	0.189	0.018	-0.114	-0.13	-0.259	0.043	0.319	-0.133	-0.039	-0.132	-0.098
**P75**	0.124	-0.036	0.2	0.092	-0.09	-0.189	-0.256	0.143	0.405	-0.119	0.031	-0.116	-0.131
**P95**	0.087	-0.086	0.11	0.043	-0.081	-0.155	-0.172	0.116	0.282	-0.08	0.019	-0.117	-0.104
**P99**	-0.002	-0.036	-0.008	-0.063	-0.079	-0.006	-0.099	-0.025	0.056	-0.079	-0.062	-0.123	-0.063
**ASM**	-0.082	0.049	-0.141	0.012	0.057	0.05	0.221	-0.246	-0.434	0.071	-0.062	-0.052	0.179
**Contrast**	-0.001	-0.072	0.01	0.009	0.007	-0.009	-0.076	0.112	0.185	-0.046	0.082	-0.051	-0.107
**Correlation**	-0.183	0.021	-0.243	-0.294	-0.131	0.191	0.175	-0.173	-0.332	0.075	-0.126	0.196	0.182
**Sum Square**	0.08	-0.019	0.17	0.051	-0.121	-0.178	-0.234	0.064	0.317	-0.121	-0.017	-0.127	-0.1
**Sum Average**	0.077	-0.002	0.18	0.047	-0.131	-0.173	-0.246	0.052	0.308	-0.128	-0.029	-0.134	-0.103
**I Difference Moment**	-0.068	0.108	-0.124	-0.045	0.087	0.125	0.198	-0.249	-0.411	0.079	-0.146	-0.028	0.151
**Sum Entropy**	-0.11	0.074	-0.13	-0.043	0.033	0.085	0.177	-0.244	-0.407	0.055	-0.06	0.063	0.162
**Entropy**	0.058	-0.08	0.091	0.017	-0.037	-0.049	-0.167	0.232	0.376	-0.064	0.09	-0.027	-0.158
**Difference Variance**	-0.001	-0.072	0.01	0.009	0.007	-0.009	-0.076	0.112	0.185	-0.046	0.082	-0.051	-0.107
**Sum Variance**	0.086	-0.025	0.177	0.055	-0.125	-0.183	-0.24	0.077	0.331	-0.121	-0.014	-0.132	-0.106
**Difference Entropy**	0.033	-0.133	0.052	0.016	-0.073	-0.102	-0.128	0.201	0.32	-0.058	0.118	-0.031	-0.113
**IMC1**	0.075	0.086	0.035	0.042	0.136	0.137	0.075	0.03	-0.054	0.103	-0.086	-0.166	-0.024

Table S 3: Analysis of error and absolute errror in the prediction of IDH status and Grade according to acquisition parameters. Groups of parameters are as follows: FA: Flip angle 0 - 90; 1 - <90; FS: Field Strength 0 - 1.5T / 1 - 3T; MAT: Matrix Acquisition 0 - < 128 X 128 / 1 - > 128X128; RES: Axial resolution 0 - ¡= 1mm X 1mm / 1 [1mm - 2mm] / 2 - > 2mm; SCAN: Scanner manufacturer 0 SIEMENS / 1 Philips / 2 GE; SEQ: Sequence acquisition 0 Spin echo / 1 Gradient Echo; SLICE: Slice thickness 0 < 5mm / 1 $\geq$ 5mm ; TE : Echo time 0 - [25 - 44] ms / 1 - [45 - 55 ms] ; TR Repetition time 0 - <1499ms / 1 - > 1500ms

		IDH					Grade				
		**Mean**	**SD**	**Mean**	**SD**	**P**	**Mean**	**SD**	**Mean**	**SD**	**P**
**Error**	**FA01**	0.038	0.467	0.052	0.495	0.789	-0.153	0.766	-0.183	0.945	0.752
	**FS01**	0.022	0.431	0.072	0.533	0.352	-0.097	0.791	-0.253	0.913	0.098
	**MAT01**	0.041	0.462	0.046	0.491	0.933	-0.16	0.729	-0.17	0.923	0.919
	**RES01**	0.097	0.368	0.025	0.487	0.311	-0.053	0.713	-0.158	0.884	0.421
	**RES02**	0.097	0.368	0.076	0.541	0.818	-0.053	0.713	-0.317	0.808	0.081
	**RES12**	0.025	0.487	0.076	0.541	0.503	-0.158	0.884	-0.317	0.808	0.237
	**SCAN01**	0.058	0.495	0.065	0.467	0.916	-0.201	0.893	-0.247	0.742	0.683
	**SCAN02**	0.058	0.495	-0.031	0.442	0.218	-0.201	0.893	0.065	0.821	**0.044**
	**SCAN12**	0.065	0.467	-0.031	0.442	0.217	-0.247	0.742	0.065	0.821	**0.019**
	**SEQUENCE01**	0.042	0.491	0.046	0.467	0.934	-0.188	0.898	-0.141	0.792	0.62
	**SLICE01**	0.177	0.539	0.018	0.463	**0.027**	-0.264	0.835	-0.147	0.852	0.357
	**TE01**	0.05	0.477	-0.042	0.511	0.408	-0.177	0.854	0.001	0.769	0.366
	**TR01**	0.046	0.488	0.038	0.45	0.895	-0.171	0.893	-0.151	0.689	0.857
**Absolute error**	**FA01**	0.282	0.374	0.333	0.37	0.214	0.524	0.58	0.712	0.647	**0.006**
	**FS01**	0.256	0.348	0.366	0.395	**0.008**	0.54	0.586	0.693	0.646	**0.024**
	**MAT01**	0.266	0.38	0.33	0.366	0.123	0.477	0.574	0.695	0.631	**0.001**
	**RES01**	0.196	0.326	0.32	0.367	**0.024**	0.438	0.565	0.648	0.622	**0.026**
	**RES02**	0.196	0.326	0.346	0.422	**0.045**	0.438	0.565	0.603	0.624	0.161
	**RES12**	0.32	0.367	0.346	0.422	0.656	0.648	0.622	0.603	0.624	0.644
	**SCAN01**	0.324	0.378	0.275	0.382	0.33	0.66	0.635	0.508	0.595	0.062
	**SCAN02**	0.324	0.378	0.287	0.338	0.506	0.66	0.635	0.587	0.577	0.437
	**SCAN12**	0.275	0.382	0.287	0.338	0.854	0.508	0.595	0.587	0.577	0.429
	**SEQUENCE01**	0.32	0.374	0.287	0.371	0.423	0.662	0.635	0.544	0.592	0.083
	**SLICE01**	0.386	0.415	0.289	0.363	0.079	0.6	0.638	0.609	0.614	0.927
	**TE01**	0.303	0.372	0.329	0.394	0.767	0.615	0.619	0.492	0.59	0.392
	**TR01**	0.315	0.376	0.268	0.364	0.334	0.656	0.63	0.446	0.547	**0.009**

Overall

	**IDH - wildtype**	**IDH-mutant**
**IDH-wildtype**	143 (43%)	39 (12%)
**IDH-mutant**	53 (16%)	98 (29%)

1.5T

	**IDH-wildtype**	**IDH-mutant**
**IDH-wildtype**	91 (49%)	19 (10%)
**IDH-mutant**	23 (12%)	53 (29%)

3T

	**IDH-wildtype**	**IDH-mutant**
**IDH-wildtype**	52 (35%)	20 (14%)
**IDH-mutant**	30 (20%)	45 (31%)

	**II**	**III**	**IV**
**II**	43(11%)	27(8%)	31(10%)
**III**	18(5%)	17(5%)	39(12%)
**IV**	11(4%)	30(8%)	117(35%)

	**II**	**III**	**IV**
**II**	24(13%)	10(5%)	13(7%)
**III**	10(5%)	11(6%)	22(12%)
**IV**	4(2%)	1810%	74(40%)

	**II**	**III**	**IV**
**II**	19(13%)	17(12%)	18(12%)
**III**	8(5%)	6(4%)	17(12%)
**IV**	7(5%)	12(8%)	43(29%)

Table S 4: Confusion matrix over different field strengths for the mutation status prediction(top row) and the grade prediction(bottom row)

Table S 5: For all error types, mean z-score difference between the erroneously classified elements and the rightly classified elements for each feature corrected for acquisition parameters. Bold fonts correspond to cases where the difference reached significance for Wilcoxon two sample test (p¡0.05).

	**IDH instead of WT**	**WT instead of IDH**	**2 instead of 3**	**2 instead of 4**	**3 instead of 4**	**3 instead of 2**	**4 instead of 2**	**4 instead of 3**
Vol	12476.119	-7444.719	-12537.293	11188.493	3182.302	-2241.8	13697.32	-5260.929
Surface	1848.966	-528.481	-4318.141	333.778	361.377	797.713	**4817.192**	-140.714
SAV	-0.012	**0.022**	-0.034	-0.038	-0.002	**0.039**	**0.035**	0.011
NonCompactness	0.151	1.672	**-6.581**	-4.243	0.298	**3.689**	**7.774**	1.963
Mean	**-0.611**	**0.514**	-0.131	**-0.513**	**-0.628**	0.095	**0.511**	**0.392**
Skewness	**0.572**	**-0.417**	0.104	**0.578**	**0.74**	-0.132	**-0.659**	**-0.519**
Kurtosis	**1.915**	**-2.779**	0.323	**1.355**	**1.895**	-0.226	**-3.151**	**-3.967**
Min	**-0.186**	**0.217**	-0.096	-0.098	-0.101	**0.239**	0.212	0.091
Max	0.227	0.395	-0.283	0.481	-0.146	-0.228	0.333	-0.151
STD	**-0.19**	0.09	0	-0.094	**-0.261**	-0.041	0.079	**0.155**
P1	**-0.203**	**0.197**	-0.06	-0.09	-0.137	0.168	0.16	0.053
P5	**-0.254**	**0.276**	-0.103	-0.151	**-0.186**	0.117	**0.237**	0.076
P35	**-0.431**	**0.421**	**-0.137**	**-0.402**	**-0.367**	0.103	**0.408**	**0.223**
Median	**-0.613**	**0.543**	-0.151	**-0.595**	**-0.623**	0.107	**0.548**	**0.392**
P75	**-0.808**	**0.644**	-0.13	**-0.707**	**-0.893**	0.107	**0.679**	**0.588**
P95	**-0.977**	**0.648**	-0.094	-0.613	**-1.118**	0.028	**0.615**	**0.696**
P99	**-0.771**	**0.538**	-0.213	-0.225	**-0.902**	-0.125	0.387	0.474
ASM	-0.005	**-0.005**	0.003	0	0.001	0.004	0.025	-0.005
Contrast	**-10.093**	2.896	-0.587	-7.286	**-10.813**	1.185	4.027	7.328
Correlation	**6.295**	**-3.713**	0.691	**4.624**	**6.588**	-2.301	**-5.383**	**-4.937**
Sum Square	**-641.895**	**498.674**	-113.993	**-541.43**	**-679.392**	75.033	**496.735**	**403.621**
Sum Average	**-11.833**	**10.016**	-2.735	**-10.092**	**-12.134**	1.89	**9.993**	**7.673**
I Different Moment	**0.068**	**-0.061**	0.039	**0.07**	**0.074**	0.003	**-0.053**	**-0.058**
Sum Entropy	**0.205**	**-0.189**	0.054	**0.19**	**0.274**	0.066	**-0.117**	**-0.251**
Entropy	**0.441**	**-0.401**	0.197	**0.412**	**0.542**	0.095	**-0.324**	**-0.451**
Difference Variance	**-0.101**	0.029	-0.006	-0.073	**-0.108**	0.012	0.04	0.073
Sum Variance	**-2647.846**	**2077.71**	-505.909	**-2260.382**	**-2803.464**	321.106	**2079.12**	**1679.264**
Difference Entropy	**-0.176**	**0.122**	-0.102	-0.152	**-0.198**	-0.004	**0.099**	**0.131**
IMC1	-0.02	-0.017	**0.031**	0.006	-0.019	-0.014	0.061	0.009

Supplementary Figures

Supplementary Figure S1 gives the details of the DSC-MRI acquisition technical parameters and scanner specifications. DSC-MRI was performed as part of the standard clinical care in newly diagnosed presumed gliomas. The conventional imaging across the participating centres included 3-plane localizers, sagittal spin-echo (SE) T1-weighted images, axial 3D T1-weighted gradient-echo (GRE) images before and after gadolinium injection, axial DWI, axial and coronal T2- FLAIR, and axial FSE T2-weighted sequences. DSC-PWI was obtained using both SE and GRE techniques with their parameters summarized as follows: slice thickness 4 - 5 mm, matrix size 96 x 96 - 128 x 128, FOV 200 230 mm, TR: 1025 1878 ms, TE: 30 45 ms, flip angle: 25 90. Each perfusion series consisted of 60-80 dynamic images in all acquisition settings, and the total acquisition time ranged from 111- 190 seconds. The gadolinium contrast agents included Magnevist (Berlex, Montville, NJ, USA), Gadovist (Bayer AG, Leverkusen, Germany), Dotarem (Guerbet SA, Villepinte, France) and Prohance (Bracco Diagnostic Inc., Monroe Township, NJ, USA) and were injected using the respective body weight adapted scheme suggested by the vendor. The bolus was injected using in all cases a power injector with an injection rate of 3-5 mL/s, followed by administration of 20-mL bolus of saline at the same injection rate. All patients had received pre-bolus saturation using either half or full dose of gadolinium.

FS	SCAN	SEQ	TR	TE	FA	MA	SL	RES
FS 1 186	Scan 1 86	Seq1 85	TR1 66	TE1 65	FA1 38	MA1 23	SL1 18	RES1 16
								RES2 2
							SL2 5	RES1 2
								RES3 3
						MA2 15	SL1 1	RES2 1
							SL2 14	RES1 7
								RES2 7
					FA2 27	MA2 27	SL2 27	RES1 1
								RES2 26
				TE2 1	FA2 1	MA2 1	SL2 1	RES2 1
			TR2 19	TE2 6	FA1 6	MA1 5	SL2 5	RES1 5
						MA2 1	SL1 1	RES2 1
				TE2 13	FA1 13	MA1 13	SL1 3	RES1 3
							SL2 10	RES1 9
								RES2 1
		Se2 1	TR2 1	TE2 1	FA2 1	MA2 1	SL2 1	RES2 1
	Scan 2 41	Se2 41	TR1 1	TE1 1	FA1 1	MA1 1	SL2 1	RES2 1
			TR2 40	TE1 40	FA1 40	MA1 40	SL2 1	RES1 1
							SL2 39	RES2 39
	Scan 3 59	Se2 59	TR1 59	TE1 59	FA1 1	MA2 1	SL2 1	RES2 1
					FA2 58	MA2 58	SL2 58	RES2 58
FS 3T 147	Scan1 101	Se1 93	TR1 85	TE1 80	FA1 32	MA2 32	SL1 4	RES2 4
							SL2 28	RES2 28
					FA2 48	MA2 48	SL1 12	RES2 12
							SL2 36	RES1 1
								RES2 35
				TE2 5	FA1 1	MA2 1	SL2 1	RES2 1
					FA2 4	MA2 4	SL1 2	RES2 2
							SL2 2	RES2 2
			TR2 8	TE1 8	FA1 7	MA1 6	SL2 6	RES3 6
						MA2 1	SL1 1	RES2 1
					FA2 1	MA2 1	SL1 1	RES2 1
		Se2 8	TR1 1	TE1 1	FA2 1	MA2 1	SL1 1	RES1 1
			TR2 7	TE1 7	FA2 7	MA2 7	SL1 7	RES1 7
	Scan2 46	Se2 46	TR1 44	TE1 44	FA1 44	MA1 44	SL1 1	RES3 1
							SL2 43	RES2 1
								RES3 42
			TR2	TE1	FA1	MA1 2	SL1 2	RES2 2

FS: Field Strength
1- 1.5T / 2 – 3T

SCAN
1 SIEMENS
2 Philips
3 GE

SEQUENCE
1 – SE
2 – GRE

TR
1 <=1499ms
2 >=1500,s

TE
1 = [25 – 44] ms
2 = [45 – 55] ms

FA – Flip angle
1 = 90
2 <= 90

MA – Matrix acquisition
1 <= 127 x 127
2 >= 128 x 128

SL – Slice thickness
1 <= 4.99 mm
2 >= 5mm

RES – Resolution
1 <= 1mm
2 =]1 – 2[mm
3 >= 3mm

Figure S 1: Representation of the acquisition parameters repartition across the 333 used cases.
